# LEISURE ACTIVITIES, LIFE SATISFACTION, HAPPINESS, AND HEALTH PERCEPTION OF OLDER KOREAN IMMIGRANTS

**DOI:** 10.1093/geroni/igad104.3024

**Published:** 2023-12-21

**Authors:** Juwon Lee, Yongseop Kim, Jaehyun Kim, Junhyoung Kim

**Affiliations:** Henry M Gunn High School, Palo Alto, California, United States; Indiana University, Bloomington, Indiana, United States; East Carolina University, Greenville, North Carolina, United States; Indiana University, Bloomington, Indiana, United States

## Abstract

The number of Korean immigrants in the United States has been increasing rapidly, with many facing psychological challenges due to cultural differences, limited resources, and language barriers. These challenges can lead to increased stress, which negatively affects mental health. However, research has shown that leisure activities can have positive effects on health outcomes, particularly for older adults. This study aimed to investigate the relationships among leisure activities, life satisfaction, happiness, and health perception in older Korean immigrants. Convenient sampling was used to recruit 51 Korean adults aged 50-85 from community centers and churches. Results showed that participation in leisure time physical activity (LTPA) predicted life satisfaction and happiness, with outdoor physical activity being positively associated with psychological well-being. Furthermore, education was significantly related to life satisfaction and health perception among those who engaged in LTPA. The findings suggest that LTPA may be linked to higher levels of happiness, life satisfaction, and health perception in older Korean immigrants. Therefore, offering recreational programs for Korean adults in the United States could potentially improve their overall well-being. By providing opportunities for older Korean immigrants to engage in leisure activities, such as outdoor physical activity, their mental and physical health may be positively impacted. These findings contribute to the growing body of research on the importance of leisure activities for older adults’ well-being and highlight the potential benefits of providing recreational programs for minority populations.

